# The Mechanism of the Neuroprotective Effect of Kynurenic Acid in the Experimental Model of Neonatal Hypoxia–Ischemia: The Link to Oxidative Stress

**DOI:** 10.3390/antiox10111775

**Published:** 2021-11-05

**Authors:** Ewelina Bratek-Gerej, Apolonia Ziembowicz, Jakub Godlewski, Elzbieta Salinska

**Affiliations:** 1 Department of Neurochemistry, Mossakowski Medical Research Institute, Polish Academy of Sciences, 02-106 Warsaw, Poland; aziembowicz@imdik.pan.pl (A.Z.); elasalin@gmail.com (E.S.); 2 Tumor Microenvironment Laboratory, Mossakowski Medical Research Institute, Polish Academy of Sciences, 02-106 Warsaw, Poland; jgodlewski@imdik.pan.pl

**Keywords:** neonatal hypoxia–ischemia, kynurenic acid (KYNA), oxidative stress, neuroprotection

## Abstract

The over-activation of NMDA receptors and oxidative stress are important components of neonatal hypoxia–ischemia (HI). Kynurenic acid (KYNA) acts as an NMDA receptor antagonist and is known as a reactive oxygen species (ROS) scavenger, which makes it a potential therapeutic compound. This study aimed to establish the neuroprotective and antioxidant potential of KYNA in an experimental model of HI. HI on seven-day-old rats was used as an experimental model. The animals were injected i.p. with different doses of KYNA 1 h or 6 h after HI. The neuroprotective effect of KYNA was determined by the measurement of brain damage and elements of oxidative stress (ROS and glutathione (GSH) level, SOD, GPx, and catalase activity). KYNA applied 1 h after HI significantly reduced weight loss of the ischemic hemisphere, and prevented neuronal loss in the hippocampus and cortex. KYNA significantly reduced HI-increased ROS, GSH level, and antioxidant enzyme activity. Only the highest used concentration of KYNA showed neuroprotection when applied 6 h after HI. The presented results indicate induction of neuroprotection at the ROS formation stage. However, based on the presented data, it is not possible to pinpoint whether NMDA receptor inhibition or the scavenging abilities are the dominant KYNA-mediated neuroprotective mechanisms.

## 1. Introduction 

Despite recent advances in neonatology, perinatal asphyxia (hypoxia–ischemia), remains a common problem in clinical practice, occurring in 2–4 per 1000 live births at term [1]. While acute asphyxia occurs in 1 per 1000 live births, mild hypoxia ensues more frequently, affecting up to 2% of all childbirths [2,3]. A hypoxic–ischemic (HI) insult is the result of a transient or permanent interruption of the blood and, thus, the oxygen supply to the infant’s brain during delivery [4].

Severe asphyxia results in multi-organ failure and may lead to perinatal fatalities [5]. The most significant problem from a healthcare standpoint is children with a medium level of HI encephalopathy, which often leads to severe developmental disorders, including cerebral palsy, cognitive impairment, and/or epilepsy [6]. The standard-of-care treatment for asphyxiated neonates remains unsatisfactory, and, despite applied procedures, traces of neonatal asphyxia remain in the form of lifelong neurological and intellectual deficits. While some therapeutic avenues to prevent asphyxia-related complications in full-term neonates (e.g., hypothermia) exist, pharmacological approaches are still lacking [7]. Therefore, the development of effective and safe drug regimens that could be used in humans in various forms of brain ischemia, including birth asphyxia, is a subject of research efforts. 

The accumulation of reactive oxygen species (ROS) is one of the most important factors involved in HI brain injury. An increased concentration of ROS shifts the antioxidant/oxidant balance towards the latter, thus initiating oxidative stress. Cells are equipped to combat oxidative stress and to neutralize ROS; superoxide dismutase (SOD), catalase (CAT), and glutathione peroxidase (GPx) supported by glutathione are well-known antioxidant enzymes [8]. However, in HI conditions, this antioxidant cell defense often fails.

HI induces the over-activation of many proteins, including hypoxia-induced factor-1α (HIF-1α). HIF-1α translocation to the nucleus stimulates pro-apoptotic genes, including the Bcl-2 family and apoptosis-inducing factor (AIF), but also the expression of sentinel proteins, such as poly (ADP-ribose) polymerase-1 (PARP-1), inducing further injury [9].

The immature brain displays high excitability, mostly due to a developmental increase in the expression of NMDA and α-amino-3-hydroxy-5-methyl-isoxazole-4-propionic acid (AMPA) receptors, and their different subunit composition when compared to the adult brain [10]. Moreover, the neonatal brain is vulnerable to oxidative stress damage due to lower levels of antioxidants. These differences account for differential responses to anti-ischemic insult treatments. A variety of inhibitors of NMDA receptors have been shown to have protective effects in adult hypoxic ischemia. However, it was found that the application of the NMDA receptor antagonist in neonates may cause abnormal neurodegeneration because activation of the NMDA receptor is required for the normal development of the brain [11,12]. Therefore, the safety and long-term effects of applying NMDA receptor antagonists for HIE treatment require a factor that will block receptors for a limited time.

Kynurenic acid (KYNA), an endogenous molecule produced in the kynurenine pathway of tryptophan metabolism, is known as an endogenous modulator of glutamatergic and cholinergic neurotransmission [13,14,15]. Its involvement in mechanisms initiated by ischemia was signalized by the information that the level of KYNA in the brain and cerebrospinal fluid increases significantly, but only for a short time after ischemia, and that its distribution matches the infarct brain regions [16,17]. 

KYNA is an endogenous antagonist of NMDA receptors binding to the glycine site [18], and this KYNA property was implied to exert neuroprotection in various preclinical models of hypoxic–ischemic brain injury [19,20,21]. However, the involvement of other KYNA properties, such as anti-inflammatory and receptor-independent anti-oxidative properties, in neuroprotection has not been studied [22,23].

KYNA, when administered peripherally, penetrates the blood–brain barrier, thus being a promising therapeutic agent to decrease excitotoxicity [24]. Another advantage of KYNA is that it cannot be metabolized to excitotoxic agents, and scavenges oxygen radicals, decreasing cellular damage [25]. The application of KYNA in high concentrations or for a prolonged time causes damage to neuronal cells [26,27]; however, KYNA applied in small doses is quickly removed from the organism and only accumulates in the liver [28]. 

This study aimed to establish the neuroprotective and antioxidant potential of KYNA administered in small doses in an experimental model of birth asphyxia. 

## 2. Materials and Methods 

### 2.1. Ethics Approval and Consent for Participation 

All described experiments were conducted according to the guidelines of the Declaration of Helsinki, approved by the 4th Local Ethical Committee (263/2017) based in Warsaw, Poland, and were performed following Polish governmental regulations (Dz.U.97.111.724), the European Community Council Directive of 24 November 1986 (86/609/EEC) and Directive 2010/63/EU. 

The animals came from breeding run by the Mossakowski Medical Research Institute’s animal facility. Each experiment was performed on 3 different litters (10–12 rats per litter), and animals were randomly selected for experimental groups (2–3 animals from each litter). All surgeries were performed under anesthesia, and all efforts were made to minimize animal suffering and the number of animals used. The mortality rate did not exceed 5%.

### 2.2. Experimental Hypoxia–Ischemia on 7-Day-Old Rats

Hypoxia–ischemia (H-I) was induced according to Rice et al. [29] with small modifications [30]. Briefly, seven-day-old Wistar rat pups of both sexes were anesthetized with sevoflurane. The left common carotid artery was exposed and cut between double ligatures of silk sutures, or was only exposed (sham control). After 60 min of recovery, the pups were placed for 75 min in a humidified chamber filled with a hypoxic gas mixture (7.3% oxygen in nitrogen, 35 °C). After hypoxic treatment, the pups were returned to their cages and housed with their mothers.

### 2.3. Drug Application 

KYNA (Tocris Bioscience, Bristol, UK) (300, 150, 50 mg/kg of body weight) was administered intraperitoneally at 1 h or 6 h after HI. The doses of KYNA were determined based on our previous experiments and the literature [24]. Sham-operated and HI control rats were injected with saline.

### 2.4. Evaluation of Brain Damage

Fourteen days after the insult (at PND21), the rats were anesthetized with a lethal dose of Morbital and decapitated. The cerebral hemispheres were weighed separately and brain damage was assessed by the deficit in weight of the ipsilateral (left) hemisphere, expressed as a percentage of the weight of the contralateral hemisphere (to the nearest 0.1 mg). 

Histological assessment of brain damage was performed on brains isolated seven days after HI. Animals were anesthetized and then perfused transcardially with phosphate-buffered saline (PBS) followed by fixation solution (4% paraformaldehyde in PBS, pH 7.4). Brains were removed and postfixed for 3 h at 4 °C in the same fixing solution. Then brains were cryoprotected overnight in 30% sucrose solution, frozen on dry ice, and stored at −70 °C. Brains were cut into 20–30 µm coronal sections on a cryostat. Sections were stained with 0.5% cresyl violet according to the Nissl staining protocol for histological assessment of neuronal cell damage. The number of survived cells was counted under 200-fold magnification in the cortex in the visual field (250 µm × 250 µm) and the CA1 area of the hippocampus (100 µm in length) using AxioVision imaging software (Carl Zeiss, Aalen, Germany).

### 2.5. Tissue Preparation for Biochemical Analysis

Brain samples for biochemical analyses were collected 4 h after HI and 3 h after KYNA injections. The rats were decapitated, and the brain tissue samples containing the cerebral cortex and hippocampus were taken from both hemispheres for further examination. Tissues from the ipsilateral and contralateral hemispheres were homogenized separately in buffers appropriate for each analysis. The protein concentration of the homogenates was determined by the Bradford method and the homogenates were used for further analyses.

### 2.6. Determination of Oxidative Stress

#### 2.6.1. Determination of ROS Level 

The levels of ROS in brain hemispheres were measured using 2,7-dichlorofluorescein acetate (DCF-DA, Invitrogen Molecular Probes, Eugene, OR, USA). Brain homogenates were placed in 40 mM Tris-HCL buffer (pH 7.4) and incubated with 2.5 µM DCF-DA in a 96-well plate for 30 min at 37 °C. The DCF fluorescence was then detected with a multifunctional microplate reader (FLUOstar Omega, BMG LABTECH, Ortenberg, Germany) at 488 nm excitation and 530 nm emission wavelength. The relative fluorescence units (RFUs) of the homogenates were calculated per 1 mg of protein.

#### 2.6.2. Determination of Glutathione Concentration 

Brain tissue homogenates in 25 mM HEPES buffer (pH 7.4) containing 250 mM sucrose were centrifuged at 1000× *g* for 5 min at 4 °C. The supernatants were collected to measure the glutathione concentration using the Fluorimetric Glutathione Assay Kit (Sigma-Aldrich, St. Louis, MO, USA) according to the manufacturer’s instructions.

#### 2.6.3. Determination of Antioxidant Enzyme Activity

##### Superoxide Dismutase

Brain tissue homogenates suspended in 20 mM HEPES buffer (pH 7.2), containing 1 mM EGTA, 210 mM mannitol, and 70 mM sucrose per 1 g of tissue, were centrifuged at 1500× *g* for 5 min at 4 °C.

The supernatant was collected to determine SOD activity using the Superoxide Dismutase Assay Kit (Cayman Chemical, Ann Arbor, MI, USA) according to the instructions provided by the manufacturer. The activity of the enzyme is expressed as the number of enzymatic units per milligram of protein (U/mg protein).

##### Glutathione Peroxidase (GPx)

Brain tissue homogenates suspended in 50 mM Tris-HCl buffer (pH 7.5) containing 5 mM EDTA and 1 mM dithiothreitol (DTT) per 1 g of tissue were centrifuged at 10,000× *g* for 15 min at 4 °C. The supernatants were collected to determine GPx activity using the Glutathione Peroxidase Assay Kit (Cayman Chemical, Ann Arbor, MI, USA) according to the instructions provided by the manufacturer.

##### Catalase

Homogenates suspended in 50 mM potassium orthophosphate buffer (pH 7.0) containing 1 mM EDTA were centrifuged at 10,000× *g* for 15 min at 4 °C. The supernatants were collected for enzyme activity determination using the Catalase Assay Kit (Cayman Chemical, Ann Arbor, MI, USA) according to the instructions provided by the manufacturer.

### 2.7. Determination of HIF-1α Concentration

Brain tissue homogenates suspended in PBS were centrifuged at 5000× *g* for 5 min and assayed using a Rat HIF-1α ELISA Kit (MyBioSource Inc., San Diego, CA, USA) according to the user manual.

### 2.8. Statistical Analysis

The results are expressed as the mean ± SEM of each experimental group. Statistical analysis was performed using a one-way ANOVA test with Dunnett’s post hoc test for significant differences between groups (GraphPad Prism 5; GraphPad Software Inc., La Jolla, CA, USA). The differences were considered statistically significant when the *p*-value was less than 0.05.

## 3. Results 

### 3.1. The Effect of KYNA Application on HI-Induced Brain Damage 

**Figure 1:**
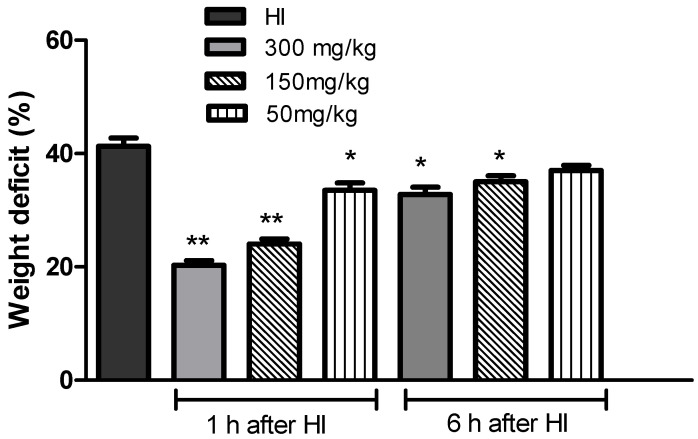
Effect of the application of KYNA on the weight deficit of the ipsilateral hemisphere after HI. KYNA (at all tested doses) was applied i.p. 1 h or 6 h after HI insult. The weight deficit is expressed as the percentage of the weight of the contralateral (right) hemisphere. The results are presented as the mean ± SEM, *n* = 4–9; * *p* < 0.05, ** *p* < 0.01 compared to the HI group.

Initially, we assessed the protective effect of KYNA on HI-induced brain damage. The HI-evoked 42% weight deficit of the ipsilateral hemisphere was significantly reduced by the application of KYNA in a dose-dependent manner 1 h after HI (*p* < 0.01) (Figure 1). A similar reduction in the weight deficit was also observed when KYNA was applied 6 h after HI, although the protective effect was less pronounced. These results highlighted the neuroprotective potential of KYNA.

**Figure 2:**
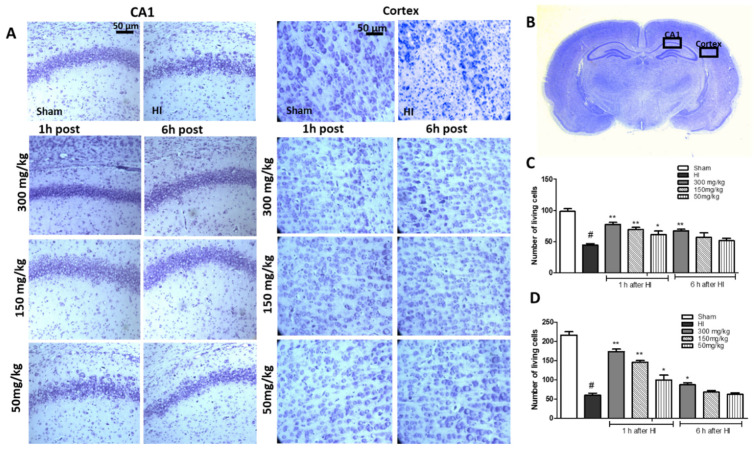
The effect of KYNA application 1 h or 6 h after HI on cell survival was observed in the CA1 region of the hippocampus and the cerebral cortex of the ipsilateral hemisphere 7 days after HI. (**A**) The microphotographs show the ipsilateral hemisphere. Scale bar represents 50 µm. (**B**) Localization of analyzed brain regions. (**C**) Quantification of surviving neurons in the central part of the CA1 region and (**D**) cortex. The results are presented as the mean ± SEM, *n* = 6; statistically significant differences: * *p* < 0.05, ** *p* < 0.01 compared to the HI group; # *p* < 0.001 compared to the sham-operated group.

Histological analysis upon HI treatment showed, expectedly, marked cell loss in the cortex, and damage and disorganization of neurons in the CA1 region of the hippocampus in the HI group (Figure 2A–C). The number of surviving neurons observed in the central part of the CA1 region was reduced by 55% (Figure 2B), and by 75.6% in the cortex (Figure 2C), compared to the control.

A detailed histological analysis of KYNA-instigated changes in the brain, which could shed more light on the KYNA neuroprotective effect, revealed that KYNA applied 1 h after HI largely prevented changes in the CA1 region of the hippocampus, and significantly decreased neuronal loss in the cortex. KYNA in a dose of 300 mg/kg, applied 6 h after HI, increased the number of surviving neurons in the CA1 region and in the cortex to 68% and 40% of the control, respectively. However, KYNA applied in lower doses did not prevent the loss of neurons in either the CA1 region of the hippocampus or in the cortex. 

The application of KYNA to sham-operated animals did not affect the weight of the brain hemispheres, and HI did not change the weight of the contralateral hemisphere (data not shown).

The results presented above clearly indicated a strong KYNA-mediated neuroprotective effect resulting from treatment 1 h after HI, and this effect was still observed when KYNA was applied 6 h later. These results defined the therapeutic window for KYNA, showing the limitations of the use of low doses.

Due to the weak neuroprotective effect of lower doses of KYNA applied 6 h after HI, these doses were not included in further analyses.

### 3.2. The Effect of KYNA Application on Changes in ROS Level in Rat Brain after HI

**Figure 3:**
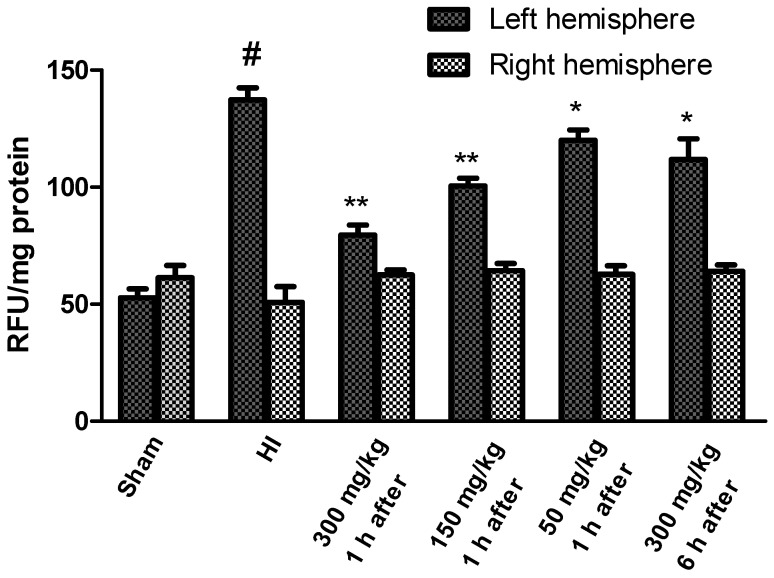
Effects of KYNA application on changes in ROS levels observed in the rat brain after HI. The results are presented as the means ± SEM, *n* = 6; statistically significant differences: * *p* < 0.05, ** *p* < 0.001 compared to the HI group; # *p* < 0.001 compared to the sham-operated group.

We then assessed the extent of KYNA-mediated alterations in the overall ROS levels to pinpoint the mechanism of KYNA-induced neuroprotection. HI increased the levels of ROS in the left ischemic hemisphere to more than 250% of the control, while remaining stable in the right hemisphere (Figure 3). KYNA treatment significantly prevented the rise in ROS levels in a dose-dependent manner, suggesting, for the first time, the mechanism beyond KYNA-mediated neuroprotection in hypoxia–ischemia. 

### 3.3. The Effect of KYNA Application on HI-Induced Changes in Antioxidant Enzymes Activity

**Figure 4:**
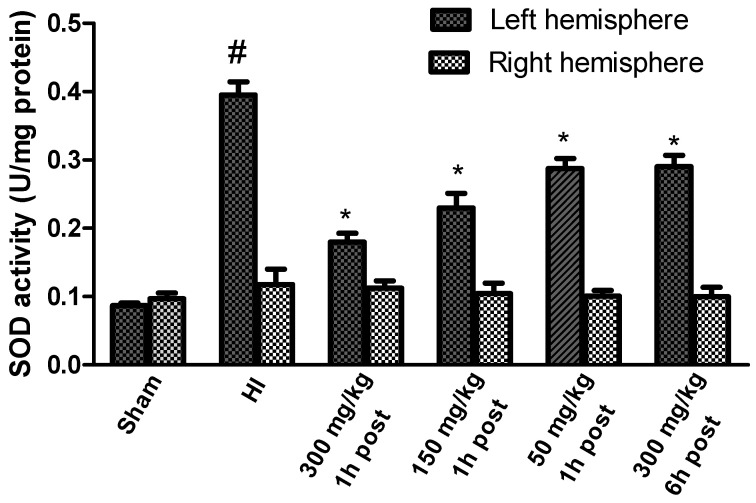
Effect of KYNA application on HI-induced changes in SOD activity. The results are presented as the means ± SEM, *n* = 6; statistically significant differences: * *p* < 0.001 compared to the HI group; # *p* < 0.001 compared to the sham-operated group.

HI increased the SOD activity to 454% of the control in the left (ipsilateral) ischemic hemisphere (Figure 4). KYNA applied 1 h after HI significantly reduced SOD activity in a dose-dependent manner. KYNA injection 6 h after HI in a dose of 300 mg/kg body weight resulted in a significant decrease in SOD activity to 333% of the control. However, the application of KYNA in doses of 50 and 150 mg/kg body weight did not result in a statistically significant decrease in SOD activity (data not shown).

HI, in this experimental model, did not change the activity of SOD in the contralateral hemisphere compared to the sham-operated group.

**Figure 5:**
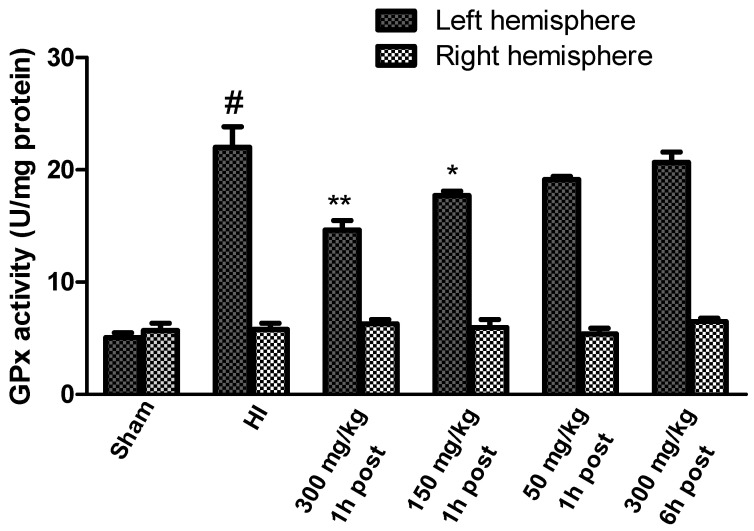
Effect of KYNA application on HI-induced changes in glutathione peroxidase activity. The results are presented as the means ± SEM, *n* = 6–7; statistically significant differences: * *p* < 0.05, ** *p* < 0.01 compared to the HI group; # *p* < 0.001 compared to the sham-operated group.

HI resulted in a significant increase in GPx activity in the left hemisphere to 440% of the control level, while the enzyme activity in the right hemisphere remained unchanged (Figure 5). KYNA in a dose of 300 mg/kg and 150 mg/kg, applied 1 after HI, significantly decreased the GPx activity in the left hemisphere to 290% and 354% of the control, respectively. KYNA in a dose of 50 mg/kg also reduced the activity of GPx (to 380% of the control), although this decrease was not statistically significant. KYNA injected 6 h after HI did not result in a decrease in GPx activity.

**Figure 6:**
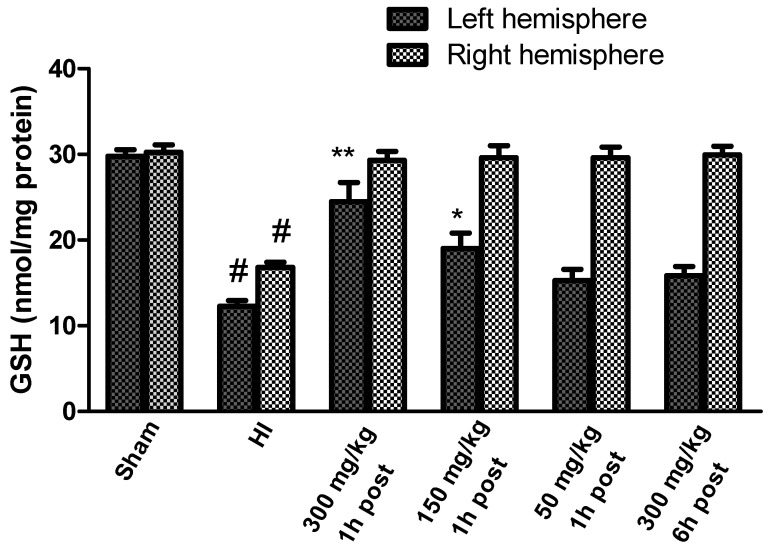
Effect of KYNA application on changes in the GSH concentration in the brains of rat pups after HI. The results are presented as the means ± SEM, *n* = 5–6; statistically significant differences: * *p* < 0.01, ** *p* < 0.001 compared to the HI group, # *p* < 0.001 compared to the sham-operated group.

The GSH concentration determined in brains isolated from control rats ranged between 29.7 and 30.25 nmol/mg of protein in the left and right hemispheres, respectively (Figure 6). 

HI resulted in a significant decrease in GSH in both hemispheres to 41.3% and 55.5% of the control in the left and right hemispheres, respectively (Figure 6). KYNA applied in doses of 300 mg/kg and 150 mg/kg body weight 1 h after HI significantly restored the GSH concentration, whereas the application of KYNA in a dose of 50 mg/kg body weight did not result in a significant increase in GSH concentration compared to the HI group, similarly to 300 mg/kg applied 6 h after HI. However, KYNA in each of the investigated doses restored GSH concentration in the right hemisphere to the control level, independently of the time of application.

**Figure 7:**
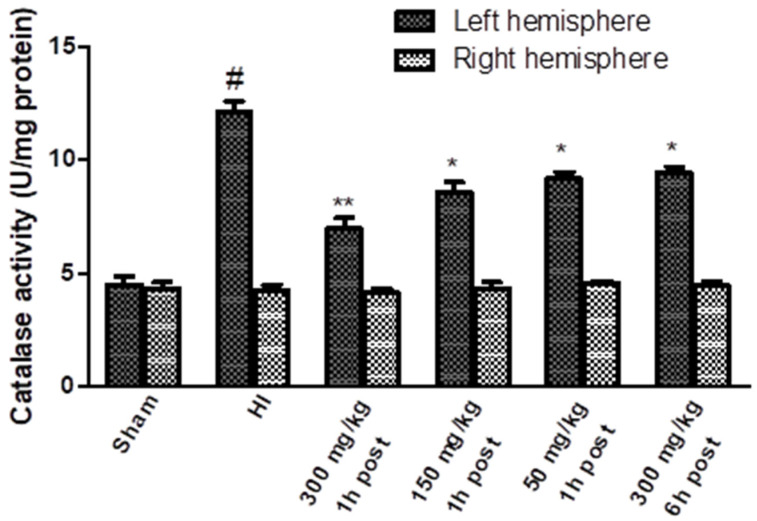
Effect of KYNA application on HI-induced changes in catalase activity. The results are presented as the means ± SEM, *n* = 6; statistically significant differences: * *p* < 0.005, ** *p* < 0.001 compared to the HI group; # *p* < 0.001 compared to the sham-operated group.

The activity of the catalase increased significantly after HI only in the left hemisphere, reaching a value of 269% of the control (Figure 7). KYNA applied 1 h after HI resulted in a significant dose-dependent decrease in catalase activity. KYNA injected 6 h after HI, in a dose of 300 mg/kg body weight, also resulted in a decrease in catalase activity to 210% of the control.

### 3.4. The Effect of KYNA Application on the Changes in HIF-1α Concentration Observed after HI

**Figure 8:**
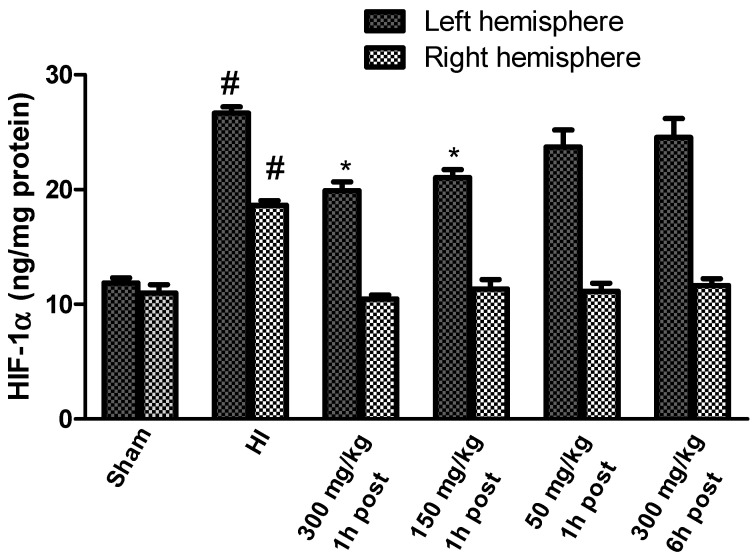
Effect of KYNA application on HI-induced changes in the HIF-1α concentration. The results are presented as the means ± SEM, *n* = 6; statistically significant differences: * *p* < 0.001, compared to the HI group; # *p* < 0.001 compared to the sham-operated group.

The HIF-1α concentration measured in the brains of the sham-operated rats ranged from 11.85 to 10.97 ng/mg protein in the left and right hemispheres, respectively. HI significantly increased the HIF-1α concentration to 26.6 ng/mg protein in the left hemisphere, and to 18.65 ng/mg protein in the right hemisphere, which is 225% and 169.5% of the control, respectively (Figure 8). The application of KYNA in a dose of 300 mg/kg and 150 mg/kg body weight 1 h after HI significantly decreased the HIF-1α concentration to 168% and 177% of the control, respectively. 

However, KYNA in a dose of 50 mg/kg body weight did not result in a statistically significant decrease in the HIF-1α concentration, and the application of KYNA in a dose of 300 mg/kg body weight 6 h after HI did not reduce the HIF-1α concentration in the left, ischemic hemisphere; however, KYNA application at all the used doses significantly reduced the HI-increased concentration of HIF-1α in the right hemisphere, restoring it to the control level.

## 4. Discussion

The results of the present study demonstrate a protective effect of the early application of KYNA on the development of neuronal injury in a rat model of perinatal asphyxia.

Our results show that the administration of KYNA in a dose of 300 mg/kg of body weight results in neuroprotection if it takes place up to 6 h after HI. KYNA in smaller doses (50 and 150 mg/kg) is only effective when applied immediately after HI. The application of KYNA 1 h after HI significantly reduced weight loss in the ischemic hemisphere, and reduced neuronal loss in the CA1 region of the hippocampus and cortex, whereas the neuroprotective effect of KYNA applied 6 h after HI was negligible. 

It was shown that after profound asphyxia, a “latent” phase develops, typically lasting approximately 6 h, during which the brain can still recover from the insult, only to die hours to days later after a “secondary” deterioration, characterized by seizures, cytotoxic edema, and progressive failure of cerebral oxidative metabolism. Therefore, accepted treatments and experimental therapies of HI should be initiated before the onset of secondary deterioration [31,32,33]. Our results indicate a short therapeutic window for KYNA treatment, which is in line with the generally accepted treatment initiation time. The observed neuroprotective effects of KYNA, expressed as a reduction in neuronal loss and brain damage, agree with previous observations [21,34]; however, KYNA application temporal boundaries have been demonstrated for the first time.

Excessive glutamate release and excitotoxic NMDA receptor activation are important mechanisms of neuronal damage during primary energy loss and the reoxygenation/reperfusion phases of HIE development. Studies have shown that pretreatment with the NMDA receptor antagonist MK-801 provided only a partially effective operation in a piglet model of HIE [35], and MK-801 had not only protective, but also toxic, effects in rat pups [36]. Therefore, the use of alternative agents inhibiting the over-excitation of NMDA receptors is of great interest. 

KYNA’s property to inhibit NMDA receptors is mainly associated with its neuroprotective action, but its effective antioxidant properties and hydroxyl radical scavenging capacity may also play a role in neuroprotection [22,37]. HI generates oxidative stress manifested by the increased generation of ROS. It was shown that key antioxidant enzymes increase their activity after HI, although the level of GSH decreases, probably as an effect of intensive consumption in a reaction catalyzed by GPx [31,38]. KYNA was shown to reduce ROS levels and regulate antioxidant enzyme activity in vivo in an experimental model of oxidative stress induced by an injection of quinolinic acid into a rat striatum, and in vitro on rat brain samples and *Xenopus laevis* oocytes by inducing the Fenton reaction [22,39]. 

Our results show, for the first time, that the application of KYNA 1 h after HI significantly decreases ROS levels and antioxidant enzyme activity. We also observed partial restoration of the GSH concentration. However, the application of KYNA 6 h after HI had a much weaker effect, again suggesting that there is only a short therapeutic window for KYNA.

Low oxygen activates hypoxia-inducible factor (HIF) transcription factors that play a dominant role in coordinating the transcriptional response to hypoxia. HIF-1α regulates a multitude of genes involved in glycolysis, inflammation, apoptosis, and proteolysis. The functional HIF-1 complex is formed by regulatory subunit-α (HIF-1α) and a constitutively expressed β-subunit [40]. Under normoxia, HIF-1α is rapidly degraded; however, in hypoxic conditions, its accumulation may lead to the activation of genes, such as *Nox2*, that encode the pro-oxidant enzyme NADPH oxidase, which is a major source of cellular ROS [41]. 

The presented results show that the application of KYNA 1 h after HI reduced the HIF-1α protein levels that were increased by HI. It is difficult to determine whether this decrease in HIF-1α is the result of a reduction in ROS production or an inhibition of NMDA receptors. ROS act as an important signal molecule on MAPK, PI3K/Act/mTOR, and NF-κB pathways, which regulate the expression of HIF-1α [42]. In hypoxic conditions, increased ROS activates NF-κB, which plays a key role in the transcription of HIF-1α. Moreover, it was shown that reactive nitrogen species (RNS) formed by endogenous ROS and NO inactivate HIF prolyl 4-hydroxylases (PHDs) that degrade HIF-1α [42]. On the other hand, the inhibition of NMDA receptors by MK-801 was shown to inhibit HIF-1α expression, suggesting that this mechanism may also be involved in the operation of KYNA [43]. The lack of an effect of KYNA applied 6 h after HI on the HIF-1α level again indicates the short therapeutic window for KYNA. 

## 5. Conclusions

KYNA-mediated neuroprotection observed in the HI model of birth asphyxia is, in large, connected with the reduction in oxidative stress.

KYNA reduces ROS production, and the presented results indicate that this reduction is not the result of the antioxidant enzymes’ mobilization. This suggests that the induction of neuroprotection at the ROS formation stage could be the result of KYNA’s ability to inhibit NMDA receptors and prevent calcium-induced mechanisms, leading to mitochondrial damage. However, it can also be the result of the scavenging abilities of KYNA and the direct reduction in produced ROS. Unfortunately, based on the presented data, it is not possible to pinpoint the dominant KYNA-mediated neuroprotective mechanisms, and further investigations are required. The therapeutic effect of relatively small doses of KYNA only manifests when the application is performed a short time after HI, and this therapeutic window for KYNA fits in a commonly accepted time of intervention. The presented results demonstrate KYNA’s potential as a promising new therapeutic agent.

## Data Availability

The data presented in this study are available in this manuscript.
